# Intergenerational engagement among older people in the long-term care facility in China: a mixed-methods study protocol

**DOI:** 10.1136/bmjopen-2025-104211

**Published:** 2025-08-27

**Authors:** Hao Liu, Annie E Topping, Ping Guo

**Affiliations:** 1 Department of Nursing and Midwifery, School of Health Sciences, College of Medicine and Health, University of Birmingham, Birmingham, UK; 2 University Hospitals Birmingham NHS Foundation Trust, Birmingham, UK

**Keywords:** Social Interaction, PUBLIC HEALTH, Nursing Homes, Nursing Care

## Abstract

**Introduction:**

The ageing population in China is rapidly increasing, placing a growing demand on long-term care facilities. While these facilities provide basic medical care and daily living support, there are mounting concerns about the mental and overall well-being of older people. Intergenerational engagement, which involves interactions between older and younger generations, could be a potential solution to enhance the well-being of older people in long-term care settings. However, there is a noticeable gap in empirical evidence supporting its effects in China, especially within long-term care facilities context.

**Methods and analysis:**

Our study employs an embedded mixed-methods design, integrating quantitative measures with qualitative insights, to assess the feasibility and acceptability of a 5-week intergenerational engagement intervention in a long-term care facility in China. Quantitative data will include recruitment, retention, attendance and scale completion rates, using descriptive statistics and independent sample t-tests to compare preintervention and postintervention outcomes. Qualitative data will be gathered through individual interviews with older participants and focus groups with staff and young people and will be analysed thematically to explore themes related to intervention acceptability. Integration will be undertaken at the interpretation stage, where findings from both components will be compared and synthesised to provide a comprehensive evaluation of the intervention.

**Ethics and dissemination:**

The study received approval from the Ethical Review Committee of the University of Birmingham (ERN_1775-Jul2024). Relevant local ethical approval was also obtained from the Ethics Committee of Guangzhou Furuixin Senior Apartment in China, approved in July 2024. The findings will be disseminated in peer-reviewed journals and shared through presentations at relevant conferences and meetings.

**Trial registration number:**

ISRCTN14922432.

**Strengths and limitations of this study**

- Our study’s use of both quantitative and qualitative data provides a comprehensive understanding of the feasibility and acceptability of the intergenerational engagement (IE) intervention. This design enables findings from different data sources to validate each other, thereby enhancing the robustness of the study.
- This study includes older people, young people and long-term care facility staff, providing a holistic view of the IE. This diversity helps in understanding the perspectives and experiences of different age groups and roles within the care setting.
- The inclusion of a patient and public involvement team, comprising older people, staff, young people and parents, ensured the intervention’s feasibility, accessibility and clarity, enhancing its relevance and applicability by refining the intervention design and recruitment process.
- As a feasibility study, the sample size is relatively small, which may limit the generalisability of the findings.
- The follow-up assessment will be conducted within 7 days after the intervention, which may not capture the long-term effects of the IE intervention. Longer follow-up periods would provide more information on the sustained impact of the intervention.

## Introduction

 China is experiencing a significant demographic transition with a rapidly ageing population. The Seventh National Population Census (2021) reports 264 million individuals aged 60 or above, with 190.64 million aged 65 or older. The proportion of people aged 60 and above rose from 13.26% in 2010 to 18.70% in 2020.^1^ By 2040, China is projected to have 402 million people over 60, making up 28% of the population.^2^

Key factors driving this demographic shift include the one-child policy (1979–2015), which led to an ageing population due to fewer young people.^3^ This policy, by restricting most families to a single child, has precipitated a distinctive demographic shift termed population ageing. Additionally, longer life expectancies, which increased from 70.1 years in 1996 to 77.3 years in 2019, reflect socioeconomic progress and healthcare advancements.^4^ Concurrently, birth rates have declined from 1.88 in 2017 to 1.30 in 2020, further accelerating societal ageing.^5^ The intertwined decline in fertility and the ageing process creates a mutually reinforcing cycle, posing both opportunities and challenges for public health and socioeconomic development.^2^

China’s family structure has shifted from traditional extended families to smaller nuclear families, particularly in urban areas.^6 7^ This change has impacted family dynamics and traditional family-based elderly care. Although home care remains crucial for older people, shrinking family sizes and changing living patterns necessitate further investigation into the role of families in future eldercare systems.^8^ While Japan, Taiwan and Hong Kong have relatively comprehensive long-term care (LTC) systems, Mainland China’s LTC system is still developing.^9^ It faces challenges like insufficient home and community-based services, a shortage of LTC staff, weak quality supervision and inadequate funding.^10^ Nursing homes and care facilities are increasingly popular, with over 2 million of the 170.63 million older people in China choosing to live in one of the approximately 40 000 nursing facilities available.^11^

Chinese older people face a variety of health problems, primarily non-communicable diseases. Approximately 75% of Chinese individuals aged 60 and above were living with conditions such as cardiovascular issues, diabetes and hypertension in 2019.^2^ Up to 75.8% of older people in China suffer from more than one chronic disease, elevating their health risks.^12 13^ The complexity of health issues increases with age. Over 50% of Chinese older people with depressive symptoms have at least one chronic disease, and nearly 20% have at least two.^14^ China currently has up to 40 million older people living in disabled or semidisabled conditions.^15^ The one-child policy has led to fewer younger caregivers, escalating the demand for LTC facilities.^3^ Many older people, lacking adequate family support, experience heightened loneliness and isolation, underscoring the need for more comprehensive eldercare solutions in China.

The quality of eldercare in China’s LTC facilities varies considerably due to weak regulation, inconsistent inspections and ineffective rule enforcement.^10^ While these institutions generally satisfy older people’s basic needs, significant improvements are needed in quality care.^16^ The mental well-being of older people in these facilities is a pressing concern. A study indicates that 93.2% of nursing home residents experience mild to severe psychological issues.^17^ This elevated rate of mental health challenges may stem from a lack of emotional support from families, limited social interactions and pervasive feelings of loneliness and disorientation.^18 19^ Introducing intergenerational engagement (IE) programmes could foster interpersonal interaction and provide emotional support, potentially alleviating these mental health challenges.

IE is defined as ‘an organised initiative that brings individuals together from distinct age groups, typically older people and children or youth, to mutually benefit all participants’.^20^ IE programmes facilitate structured social interactions, promoting understanding, respect and cooperation among different age cohorts. Research highlights potential benefits such as enhanced physical and psychological health, socialisation, sense of self-worth and autonomy in older people.^21^,^23^ Our previous review demonstrates that IE can reduce depression and loneliness, improve quality of life and strengthen social bonds for older people in LTC facilities.^24^ Several senior care facilities have successfully implemented IE programmes, enriching older people through cognitive and emotional stimulation and bridging generational divides.^25^ Recognising its advantages, numerous nations are integrating IE strategies to address ageing challenges and improve the mental health of older populations.^26^,^29^ However, research on IE remains sparse in China, especially within LTC facilities context. This study aims to assess the feasibility and acceptability of IE in an LTC facility in China.

## Methods and analysis

### Study design

**Figure 1:**
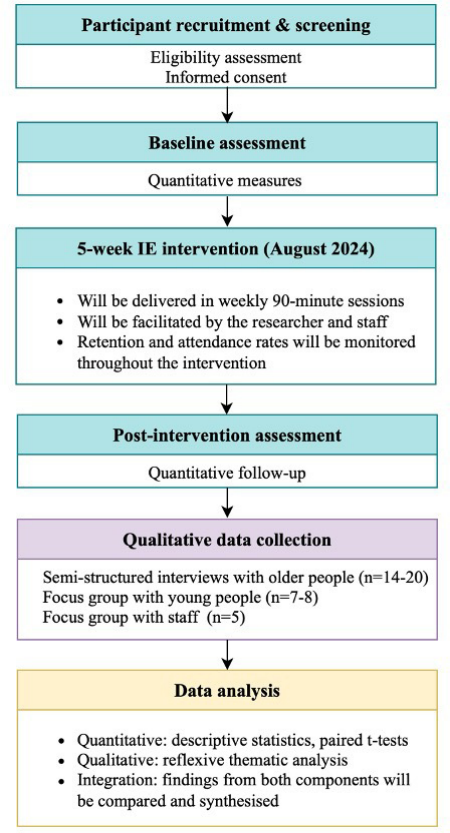
Overview of the feasibility study process. IE, intergenerational engagement.

This feasibility study employs an embedded mixed-methods design.^30^ It will integrate quantitative metrics (such as recruitment, retention, attendance and scale completion rates) with qualitative data from interviews and focus groups. Quantitative data will be analysed descriptively to assess feasibility. The interviews following translating, verification and transcription will be analysed using a reflexive thematic method^31^ to explore participants’ experiences and perceptions, providing contextual depth and informing future trial developments. Integration will be conducted at the interpretation stage, where findings from both strands will be compared and synthesised to provide a comprehensive evaluation of the feasibility and acceptability of the intervention. Figure 1 illustrates the feasibility study design. The study will be opened for recruitment in July 2024 and close following analysis in September 2025.

### Study setting

The feasibility study will be conducted at Furuixin Senior Apartment, a modern LTC facility in Guangzhou, Guangdong Province, China. This facility offers integrated medical and nursing care, with 162 available beds, rehabilitation services and a variety of amenities. When potential participants are identified, they will receive detailed information about the feasibility study, with an emphasis on the fact that participation is voluntary, and they can withdraw at any time without concerns.

### Sample size

The total sample size for this feasibility study will be 50 participants, including older people, young people and staff. Feasibility studies assess study practicality rather than intervention effectiveness or association strength, and they frequently do not include thorough power-based sample size analyses.^32 33^ Guidelines for sample sizes in feasibility studies are limited, and existing recommendations vary.^34^ In feasibility studies aimed at determining the standard deviation (SD), participant sample sizes vary; some suggest 30, while others propose a range between 24 and 50.^35^,^37^ In planning the logistics and management of the IE intervention, suggestions from patient and public involvement (PPI) members (staff) have been integral. A key consideration has been the available space in the activity room at the LTC facility. To ensure that IE activities will not be overcrowded or noisy, the feasibility study is limited by the capacity of the activity room to a maximum of 55 people. This limit will ensure ample space for both groups of participants to engage in activities while maintaining an environment conducive to interaction.

### Recruitment process

The recruitment process is scheduled to begin following ethical approval. Collaboration between the researcher, the LTC facility and the local school, with assistance from the PPI group members, will facilitate recruitment. After identifying potential participants through these collaborations, the researcher will provide detailed information and all related documents, ensuring ethical recruitment practices.

### Study participants

#### Older people

This feasibility study will recruit 30 older people residing at the study site for the IE study. Eligibility criteria for older people will include: (1) aged 60 years or above; (2) in relatively good physical and mental health, able to perform daily activities with minimal assistance and actively participate in the study without requiring intensive medical or psychological support; (3) able to communicate effectively in either Cantonese or Mandarin, including understanding, responding to questions and engaging in conversations and (4) willing to participate in the IE programme and provided signed informed consent.

Older people will be excluded if they: (1) have medium or severe cognitive impairments, experiencing an acute phase of a chronic disease, have severe physical disabilities (eg, bedbound) or were in the end-of-life period; (2) were unable to communicate effectively in either Cantonese or Mandarin and (3) declined to provide informed consent or expressed unwillingness to engage in the IE programme.

#### Young people

We will recruit 15 young people aged 15–16 years from a local school near the LTC facility. Eligibility criteria for young participants included: (1) willingness to participate in the IE programme and provision of a signed assent form, with written consent also obtained from a parent or legal guardian; (2) relatively good physical and mental health and (3) ability to communicate effectively in either Cantonese or Mandarin.

#### LTC facility staff

Five staff will be recruited. Eligibility criteria for staff will include: (1) currently employed at the LTC facility and (2) willingness to assist with the IE programme and after eliciting informed signed consent, participate in a focus group following delivery of the intervention.

### Intervention

The IE intervention was developed using insights from our previous systematic review and was guided by the activity theory of ageing,^38^ tailored to Chinese cultural contexts. Feedback from a PPI team comprising older people, LTC staff, young people and parents ensured the intervention’s feasibility, accessibility and clarity. The IE intervention will be delivered through a series of 90 min small group sessions delivered over 5 weeks The group activities will involve 2–4 older people and 1–2 young people per group. During these interactions, the researcher and staff will serve as facilitators, promoting inclusive conversations and ensuring all participants feel valued and heard. The detailed weekly activities are outlined in table 1. Each session will include warm-up games, hands-on activities, sharing of stories and experiences, and refreshments, fostering meaningful intergenerational connections.

**Table 1:** The IE programme schedule plan

	Activity	Agenda
Week 1	The charm of traditional Chinese painting	Begin the session with a warm welcome and a brief introduction to the day’s activities. Everyone introduces themselves to the group. Engage in fun ice breaker games to help participants get to know each other and feel comfortable. Start by watching a video together to learn about the basics of traditional Chinese painting. And then engage in a hands-on activity where participants practise painting in traditional Chinese fans. Enjoy some refreshments while reflecting on the activity.
Week 2	Memories of traditional festivals	Begin with a warm welcome and engaging warm-up games. Start by watching a short video introducing traditional celebrations and festivals. Older people and young people then exchange stories about their favourite traditional festivals or share interesting anecdotes about the origins of these festivals. Finally, engage in activities like lantern-making, knitting and paper cutting. Enjoy some refreshments while reflecting on the activity.
Week 3	The beauty of traditional music	Begin with a warm welcome and engaging warm-up games. Start with a brief overview of the history of traditional Chinese music, including its cultural background and evolution. Conduct some related games, such as guessing the song from its melody and a singing relay. Participants will sing some familiar traditional songs together, and then engage in a discussion to share thoughts, feelings and personal experiences related to traditional Chinese music. Enjoy some refreshments while reflecting on the activity.
Week 4	Chinese tea journey	Begin with a warm welcome and engaging warm-up games. Start with a short video introducing various Chinese teas, followed by a demonstration of traditional brewing methods. Introduce freeze-dried tea, explaining its production process and benefits. Then, set up a tea tasting session with notes to help participants identify different flavours and aromas. At the same time, participants can share personal stories and memories related to tea, including family traditions and cultural practices. Enjoy some refreshments while reflecting on the activity.
Week 5	Past and present	Begin with a warm welcome and engaging warm-up games. Show photos of various old items and ask participants to guess their uses, such as old cell phones and typewriters. Encourage older participants to share stories and experiences related to these items, providing insights into their past lives. When introducing modern cell phones, have young people demonstrate their features, highlighting the contrast between past and present technology. Then in small groups, young people will help older people use smartphone apps to create fashionable photos. Farewell party.

IE, intergenerational engagement.

### Outcomes

Aspects of feasibility will be assessed using metrics derived from the following: (1) Recruitment rate: the effectiveness of recruitment strategies will be determined by the percentage of eligible residents who agree to participate, with the response rate calculated as the number recruited compared with the number approached. (2) Retention rate: Determine the proportion of participants who remain enrolled from the start to the end of the study. Document and categorise reasons for dropout, if available. (3) Attendance rate: Calculating the ratio of sessions attended by each participant to the total number of sessions offered, often expressed as a percentage. (4) Scale completion rate (specifically for older people): Assess the completeness of scale responses by calculating the proportion of older people who fully complete the scales and noting the number of missing items per scale. Four scales will be used, including the modified Chinese version of the Geriatric Depression Scale 11-item version (CGDS-11) for measuring depression, the Chinese version of the Geriatric Anxiety Inventory (GAI-CV) for assessing anxiety, the Chinese version of the De Jong Gierveld Loneliness Scale (DJGLS) for gauging loneliness and the Chinese versions of the 12-item Short Form Health Survey (SF-12) for evaluating quality of life. All four scales have existing Chinese and English versions available.

The CGDS-11 is derived from the Chinese version of GDS-15, with questions 2, 8, 9 and 13 based on GDS-15.^39^ It is applied in a variety of healthcare and research contexts and has become known as an essential tool to assess the mental health of older people. The items of GDS use a simple yes/no response format, which is particularly preferred by older people. The Cronbach’s alpha coefficient for the CGDS-11 is 0.763, and the test–retest reliability is 0.712.^39^

The GAI-CV has strong psychological qualities, making them reliable in identifying anxiety in older people.^40^,^42^ Its predictive and concurrent validity have been verified.^43 44^ The GAI-CV shows strong reliability (Cronbach’s α=0.937) and a fitting three-factor model (Comparative Fit Index [CFI]=0.891, Root Mean Square Error of Approximation [RMSEA]=0.084), making it an effective tool for measuring anxiety among Chinese older people.^45^

The DJGLS is a reliable instrument for assessing loneliness, available in 11-item and 6-item versions that measure both emotional and social loneliness.^46^ With a Cronbach’s alpha of 0.820 for the overall scale and scores of 0.792 and 0.737 for the social and emotional loneliness subdimensions, respectively, the Chinese version of the DJGLS shows high reliability and validity, making it highly suitable for assessing loneliness among Chinese older people.^47^

The SF-12 is a shorter version of the SF-36 designed to effectively assess an individual’s quality of life.^48^ The SF-12 contains 12 questions spanning 8 health domains, adopting both Likert scales and binary choices like ‘yes’ or ‘no’.^49^ Numerous studies have shown that the Chinese version of the SF-12 is a viable and reliable instrument for assessing the health and quality of life of the Chinese elderly population.^50^,^52^

Both acceptability and feasibility will be evaluated through semistructured and focus group interviews, focusing on participants’ (residents, young people and staff) satisfaction with the IE intervention, their willingness to continue with or recommend the programme, and their experiences and perceptions of the IE intervention and suggestions for improvement.

Purposive sampling^53^ will be used to select 14–20 older people for qualitative individual interviews based on their interest in participating. This recruitment strategy emphasises selecting participants who are willing to engage in the interview process, thereby ensuring that the data collected are both rich and insightful. The interviews are designed to delve deeply into participants' experiences and perceptions with the IE intervention, with a special focus on its acceptability. Key areas of inquiry will include satisfaction with the intervention, willingness to continue or recommend, acceptability of the scales and their overall experiences and perceptions.

In addition to individual interviews with residents (older people), the study will involve two separate focus groups. One group will comprise five staff members directly involved in implementing the IE intervention. This group will provide an opportunity to discuss their observations during the intervention, the feasibility and acceptability of implementing it within the facility, and any perceived benefits or challenges. Key discussion topics will include satisfaction with the intervention, observed intergenerational interactions and suggestions for improvement.

The other group will consist of 7–8 young people who have actively participated in the IE intervention and are willing to take part in the group discussion. This focus group will centre on their experiences of interacting with older people, including what they found rewarding or challenging. Discussions will aim to understand young people’s views on the value of the IE intervention and the intergenerational relationships it fostered. Key areas of inquiry will involve their satisfaction with the programme, willingness to engage in similar future activities, suggestions for future improvement, and perceptions of intergenerational relationships and mutual understanding.

### Data collection

#### Before the intervention

Baseline demographic data will be collected from older people, including age, gender, education level, marital status, living support and health conditions. They will also invite to complete four scales: CGDS-11, GAI-CV, DJGLS and SF-12. The entire data collection process takes 35–40 min. For staff and young people, only demographic data will be collected.

#### During the intervention

We will record participant engagement, focusing on two key aspects: retention and attendance rates. Participant dropouts will be recorded, and the specific time of dropout will be noted if applicable. If participants are willing to provide reasons for leaving, these reasons will also be recorded. Also, we will document the attendance of each participant at every session, recording both their presence and the reasons for any absences.

#### After the intervention

In the quantitative component, older people will complete the following four scales as a post-test: CGDS-11, GAI-CV, DJGLS and SF-12. This will be conducted within 7 days after the intervention to assess its immediate feasibility results before potential external effects can have an impact.

In the embedded qualitative component, we will capture valuable insights into the acceptability of the intervention through interviews and focus groups. For older people, semistructured individual interviews will be conducted to explore their experiences, with each interview lasting 30–45 min. For staff and young people, focus groups will be organised, with each session expected to last between 60 and 90 min.

### Data analysis

In the quantitative component, descriptive statistics will be used to summarise the demographic information of participants and to provide an overview of the recruitment rate, retention rate, attendance rate and scale completion rate. This will involve calculating means, SD, frequencies and percentages.^54^ The scale completion rate will be assessed by determining the proportion of scales completed in full by participants. To examine the differences in outcomes before and after the intervention, independent sample t-tests will be employed to compare the change scores from the pretest to the post-test. In the qualitative component, thematic analysis will be used to analyse data collected from both qualitative interviews and focus groups.^55^ The content of interviews with older people and focus groups with staff and young people will be examined to identify themes related to the acceptability of the intervention. Findings from the quantitative and qualitative components will be brought together during interpretation to inform a comprehensive understanding of feasibility and acceptability.

## Ethics and dissemination

The study received approval from the Ethical Review Committee of the University of Birmingham (ERN_1775-Jul2024). Relevant local ethical approval was also obtained from the Ethics Committee of Guangzhou Furuixin Senior Apartment in China, approved in July 2024. The feasibility study was registered with the ISRCTN registry (ISRCTN14922432). Confidentiality and data protection measures are strictly adhered to throughout the study. The primary research paper will be submitted to peer-reviewed journals to contribute to the growing body of evidence on the use of IE in LTC facilities in China. A final report will be written and published on completion of the study. The results will be disseminated through peer-reviewed journals and shared through presentations at relevant conferences and meetings. These efforts aim to maximise the impact of the research and inform future practices in LTC facilities in China.
